# Proteomics‐Based Identification of the Pyroptosis‐Related Biomarker PCSK9 and Its Association With the Pathogenesis of Rheumatoid Arthritis

**DOI:** 10.1002/kjm2.70183

**Published:** 2026-02-10

**Authors:** Lei Wang, Hao‐Jie Chen, Ting‐Ting Wu, Xin‐Yi Shen, Ying‐Ying Gao, Yu‐Feng Yin, Tian Ren, Xin Chu, Jing Cao, Tao Cheng, Ming‐Jun Wang

**Affiliations:** ^1^ Department of Rheumatology and Immunology The First Affiliated Hospital of Soochow University Suzhou China; ^2^ Department of Emergency Medicine The First People's Hospital of Nantong Nantong China; ^3^ Department of Rheumatology and Immunology The First People's Hospital of Nantong Nantong China

**Keywords:** biomarker, NOD‐like receptor protein 3 inflammasome, PCSK9, proteomics, pyroptosis, rheumatoid arthritis

## Abstract

Rheumatoid arthritis (RA) is a common autoimmune disease, and early diagnosis is critical for effective treatment. This study aims to identify potential biomarkers related to pyroptosis through serum proteomics analysis, offering new insights for the early diagnosis of RA. We enrolled 100 participants, including 50 patients with RA and 50 healthy controls. Serum samples were collected and analyzed using high‐resolution liquid chromatography–tandem mass spectrometry (LC–MS/MS) for proteomics profiling. Differential protein expression analysis and functional annotation revealed significant upregulation of pyroptosis‐related proteins in the serum of patients with RA. Gene Ontology (GO) and Kyoto Encyclopedia of Genes and Genomes (KEGG) pathway analyses, along with protein–protein interaction (PPI) network analysis, showed that these proteins are involved in inflammation and immune pathways, particularly the activation of the NOD‐like receptor protein 3 (NLRP3) inflammasome. Enzyme‐linked immunosorbent assay (ELISA) validation confirmed a significant increase in PCSK9 levels in patients with RA, suggesting that PCSK9 may play a key role in the pathogenesis of RA. This study provides new directions for biomarker research in RA, particularly regarding the potential involvement of the pyroptosis pathway, with significant clinical application prospects.

**Abbreviations**
ACR: American College of Rheumatology
AFP: alpha‐fetoprotein
AGC: automatic gain control
ALT: alanine aminotransferase
ANOVA: analysis of variance
AST: aspartate aminotransferase
BMI: body mass index
BPs: biological processes
CC: cellular component
CEA: carcinoembryonic antigen
COG: clusters of orthologous groups
Cys: cysteine
DIA: data‐independent acquisition
DMARDs: disease‐modifying antirheumatic drugs
DTT: dithiothreitol
ELISA: enzyme‐linked immunosorbent assay
EULAR: European League Against Rheumatism
FC: fold change
FDR: false discovery rate
FT3: free triiodothyronine
FT4: free thyroxine
GCP: Good Clinical Practice
GO: gene ontology
GSDMD: gasdermin D
HbA1c: glycated hemoglobin
HDL‐C: high‐density lipoprotein cholesterol
IAM: iodoacetamide
IQR: interquartile range
KEGG: Kyoto Encyclopedia of Genes and Genomes
KOG: EuKaryotic Orthologous Groups
LC–MS/MS: liquid chromatography–tandem mass spectrometry
LDL‐C: low‐density lipoprotein cholesterol
LDLR: low‐density lipoprotein receptor
Met: methionine
MF: molecular function
NLRP3: NOD‐like receptor protein 3
PCA: principal component analysis
PCC: Pearson correlation coefficient
PPI: protein–protein interaction
QC: quality control
RA: rheumatoid arthritis
ROS: reactive oxygen species
TSH: thyroid‐stimulating hormone
UHPLC: ultra‐high‐performance liquid chromatography
WBC: white blood cell count

## 1 Introduction

Rheumatoid arthritis (RA) is a chronic, systemic autoimmune disease characterized primarily by persistent synovial inflammation, which ultimately leads to joint destruction, deformities, and functional impairments [1, 2, 3]. The global prevalence of RA is approximately 0.5%–1%, affecting millions of individuals worldwide [4]. Although the primary clinical manifestation of RA is joint symptoms, recent studies have indicated that RA is not confined to the joints but can involve multiple organ systems, including the cardiovascular, pulmonary, and renal systems, and is closely associated with various complications [5, 6, 7]. If not effectively controlled, RA can lead to long‐term physical disabilities, psychological stress [8], and a severe reduction in patients' quality of life [9] while imposing a significant economic burden on society and healthcare systems [10]. Despite substantial progress in RA research, the precise pathogenesis of the disease remains unclear. Current clinical treatment strategies primarily involve the use of combination therapies, including disease‐modifying antirheumatic drugs (DMARDs), biologics, and corticosteroids [11]. However, treatment outcomes remain suboptimal for some patients, with certain drugs demonstrating limited efficacy, significant side effects, and the potential for resistance with prolonged use [12]. Although numerous clinical treatment options exist, the heterogeneity and complexity of the disease have led to a lack of effective methods for accurately predicting disease progression and tailoring personalized therapies. Therefore, understanding the molecular mechanisms of RA and identifying new biomarkers and therapeutic targets have become critical priorities in clinical and basic research.

In recent years, pyroptosis, a novel form of programmed cell death [13], has been recognized for its critical role in various immune‐mediated diseases, particularly in the onset and progression of RA [14]. Unlike traditional apoptosis, pyroptosis is a cell death process driven by inflammasome activation, which exerts a potent pro‐inflammatory effect [15]. In the synovial tissue of patients with RA, pyroptosis is closely associated with sustained inflammatory responses, particularly in synovial fibroblasts and monocytes, where the extent of pyroptosis correlates significantly with disease activity [16]. The classical pyroptosis pathway is primarily initiated by activating the NOD‐like receptor protein 3 (NLRP3) inflammasome [17]. NLRP3 senses both exogenous and endogenous danger signals, leading to the activation of caspase‐1. Activated caspase‐1 cleaves gasdermin D (GSDMD), generating the N‐terminal domain, which possesses membrane‐permeabilizing activity, forming pores in the cell membrane. This results in the massive release of intracellular pro‐inflammatory substances, such as cytokines, triggering a cascade of immune responses [18]. Notably, releasing pro‐inflammatory cytokines, such as IL‐1β and IL‐18, further exacerbates both local and systemic inflammation in RA. Recent studies have demonstrated that pyroptosis plays a pivotal role in the pathological processes of RA. Pyroptosis‐related molecules, such as GSDMD, are significantly elevated in the serum of patients with RA and are closely associated with disease activity and the extent of joint damage.

Although research on pyroptosis has advanced, the specific mechanisms underlying its role in RA remain unclear [19]. Studies have shown that pyroptosis‐related molecules, such as GSDMD, are significantly elevated in patients with RA, with levels correlating with disease activity and the extent of joint damage [20]. Therefore, pyroptosis may serve as a potential biomarker for RA and a novel therapeutic target. Current research primarily focuses on inflammatory cytokines and immune cells, but comprehensive investigations into the role of pyroptosis in RA—particularly its molecular mechanisms related to inflammation and tissue damage—are still lacking.

Proteomics, as a powerful approach for studying the entire set of proteins and their functions within cells, tissues, or organisms, has become a core technology for uncovering disease mechanisms, identifying biomarkers, and discovering potential therapeutic targets [21]. Unlike genomics and transcriptomics, proteomics provides direct insight into proteins expressed within cells or tissues, particularly their post‐translational modifications and interactions. Researchers can identify differentially expressed proteins (DEPs) in specific disease states by analyzing proteomics data and exploring their functional roles and mechanisms. Thus, proteomics holds significant promise for RA research, particularly for high‐throughput sample analysis that can help identify early biomarkers and therapeutic targets for RA.

This study aims to use proteomics to investigate the association between pyroptosis and the pathogenesis of RA. We employed high‐throughput proteomics techniques to screen for DEPs related to RA in serum samples from both patients with RA and healthy controls, with a particular focus on proteins associated with the pyroptosis pathway. Bioinformatics analysis was conducted to explore these proteins' potential roles in RA and identify novel biomarkers for early diagnosis and disease activity monitoring.

## 2 Materials and Methods

### 2.1 Patient Selection and Grouping

This study included patients with RA hospitalized in the Rheumatology Department of the First People's Hospital of Nantong between April and June 2024. A total of 15 patients with active RA and 35 with stable RA were enrolled. Additionally, 50 healthy individuals were recruited as a control group during the same period (Table 1). The inclusion criteria were based on the 1987 American College of Rheumatology (ACR) classification criteria for RA and/or the 2010 ACR and European League Against Rheumatism (EULAR) revised classification criteria.

**TABLE 1:** Demographic and clinical characteristics of participants.

Characteristic	Healthy controls (S0, *n* = 50)	Stable RA (S2, *n* = 35)	Active RA (S1, *n* = 15)	ANOVA *p* (S0, S2, S1)	Post hoc comparisons (Tukey HSD)
Demographics					
Age (years), mean ± SD	58.64 ± 12.71	61.63 ± 15.40	62.20 ± 13.31	0.517	—
Female, *n* (%)	34 (68.0)	31 (88.6)	13 (86.7)	0.054	—
BMI (kg/m^2^), mean ± SD	23.30 ± 3.11	23.00 ± 2.86	24.00 ± 3.07	0.564	—
Disease activity					
DAS28‐CRP, mean ± SD	—	1.86 ± 0.81	3.97 ± 0.93	< 0.001	—
Disease duration (years), median (IQR)	—	5.5 (4.0, 11.0)	4.0 (3.0, 6.0)	0.476	—
Serological markers					
RF positive, *n* (%)	—	28 (80%)	13 (86.7%)	0.311	—
Anti‐CCP positive, *n* (%)	—	30 (85.7%)	14 (93.3%)	0.654	—
Inflammatory markers					
ESR (mm/h), mean ± SD	12.44 ± 5.08	28.09 ± 22.75	41.00 ± 25.02	< 0.001	S1 > S0, S2 > S0
CRP (mg/L), mean ± SD	2.14 ± 1.53	10.03 ± 14.93	13.73 ± 17.03	< 0.001	S1 > S0, S2 > S0
Ferritin (ng/mL), mean ± SD	183.76 ± 84.15	234.14 ± 149.44	201.60 ± 116.41	0.146	—
Routine biochemistry					
WBC (×10^9^/L), mean ± SD	6.08 ± 1.50	5.93 ± 2.54	6.91 ± 3.39	0.352	—
Neutrophils (%), mean ± SD	55.40 ± 6.61	63.12 ± 16.21	67.68 ± 13.69	0.001	S1 > S0, S2 > S0
Lymphocytes (%), mean ± SD	35.72 ± 6.45	24.59 ± 12.00	24.24 ± 13.31	< 0.001	S1 < S0, S2 < S0
Hemoglobin (g/L), mean ± SD	141.90 ± 11.14	116.61 ± 11.17	109.73 ± 20.23	< 0.001	S1 < S0, S2 < S0
Platelets (×10^9^/L), mean ± SD	243.46 ± 46.58	221.03 ± 78.50	260.60 ± 95.87	0.128	—
ALT (U/L), mean ± SD	18.06 ± 7.13	18.43 ± 11.13	18.10 ± 10.38	0.983	—
AST (U/L), mean ± SD	18.40 ± 4.31	19.63 ± 11.07	18.12 ± 5.77	0.712	—
Creatinine (μmol/L), mean ± SD	63.98 ± 10.51	64.14 ± 18.17	57.76 ± 10.73	0.266	—
Uric acid (μmol/L), mean ± SD	304.43 ± 63.78	282.80 ± 71.64	239.69 ± 101.72	0.012	S2 < S0
Fasting glucose (mmol/L), mean ± SD	5.15 ± 0.93	5.73 ± 1.43	6.08 ± 1.44	0.013	S1 > S0, S2 > S0
HbA1c (%), mean ± SD	4.96 ± 0.30	5.68 ± 1.19	5.40 ± 0.58	< 0.001	S1 > S0
Lipid profile (mmol/L), mean ± SD					
Total cholesterol	4.68 ± 0.90	5.60 ± 1.68	5.41 ± 1.43	0.005	S1 > S0, S2 > S0
Triglycerides	1.61 ± 1.44	1.47 ± 1.01	1.24 ± 0.69	0.576	—
LDL‐C	3.01 ± 0.69	3.09 ± 1.61	2.89 ± 1.21	0.857	—
HDL‐C	1.43 ± 0.32	1.56 ± 0.46	1.64 ± 0.41	0.123	—
CEA (ng/mL), mean ± SD	2.60 ± 1.41	2.06 ± 0.96	2.37 ± 1.93	0.199	—
AFP (ng/mL), mean ± SD	3.06 ± 1.73	3.59 ± 2.22	2.95 ± 1.36	0.362	—
FT3 (pmol/L), mean ± SD	4.68 ± 0.70	4.47 ± 0.67	4.70 ± 0.75	0.341	—
FT4 (pmol/L), mean ± SD	15.87 ± 2.50	15.76 ± 2.39	16.77 ± 1.63	0.355	—
TSH (mIU/L), mean ± SD	2.73 ± 1.51	2.76 ± 1.71	3.12 ± 1.81	0.716	—
Immunoglobulins and complement, mean ± SD					
IgA (g/L)	2.50 ± 0.86	3.44 ± 2.38	3.41 ± 0.93	0.015	S1 > S0
IgG (g/L)	13.05 ± 2.37	15.28 ± 3.45	16.45 ± 9.43	0.013	S1 > S0, S2 > S0
IgM (g/L)	1.22 ± 0.38	1.51 ± 1.08	1.66 ± 1.24	0.118	—
C3 (g/L)	1.12 ± 0.22	1.32 ± 0.36	1.38 ± 0.35	0.001	S1 > S0, S2 > S0
C4 (g/L)	0.24 ± 0.07	0.28 ± 0.25	0.29 ± 0.21	0.398	—
Lymphocyte subsets, mean ± SD					
CD3^+^	69.04 ± 6.96	70.73 ± 11.01	69.14 ± 10.92	0.687	—
CD4^+^	42.50 ± 3.39	39.77 ± 13.84	42.33 ± 7.40	0.363	—
CD8^+^	25.90 ± 6.61	28.53 ± 9.64	26.80 ± 8.31	0.334	—
CD16^+^CD56^+^ (NK cells)	13.38 ± 5.29	10.56 ± 6.90	9.31 ± 8.55	0.041	S1 < S0, S2 < S0
CD19^+^ (B cells)	13.94 ± 4.45	17.17 ± 5.60	17.79 ± 6.78	0.006	S1 > S0, S2 > S0
Medications, *n* (%)					
Methotrexate	—	28 (80.0%)	11 (73.3%)	0.713	—
Biologics	—	8 (22.9%)	6 (40.0%)	0.304	—
Glucocorticoids	—	20 (57.1%)	10 (66.7%)	0.529	—

*Note:* Data are presented as mean ± standard deviation or number (percentage), unless otherwise specified. Active RA was defined as DAS28‐CRP > 3.2; stable RA as DAS28‐CRP ≤ 3.2. *p* values were derived from one‐way ANOVA for continuous variables and from chi‐square or Fisher's exact test for categorical variables. Post hoc pairwise comparisons were performed using Tukey's honest significant difference (HSD) test; only statistically significant differences (*p* < 0.05) are indicated in the “Post hoc Comparisons” column.

**FIGURE 1:**
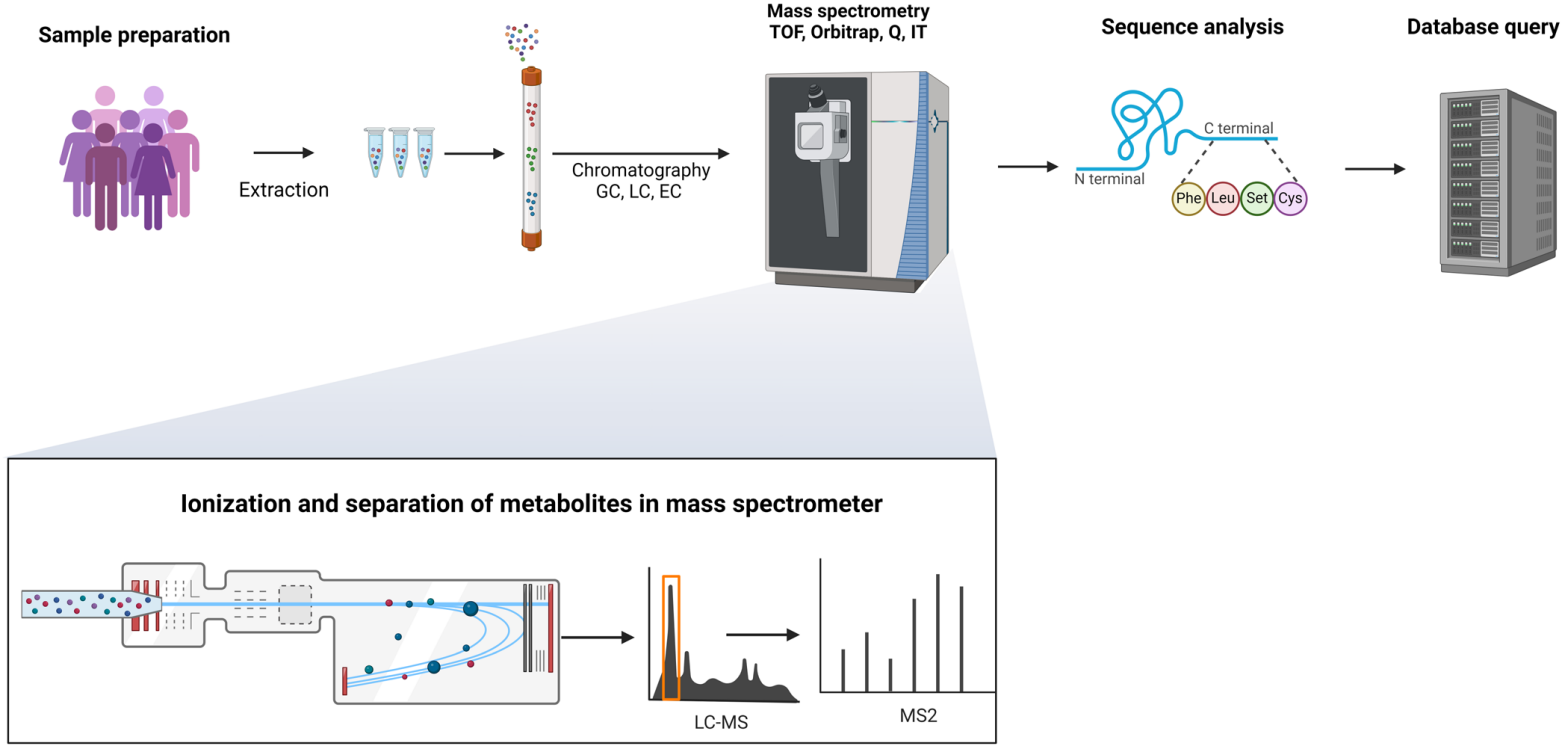
Proteomics analysis workflow.

Exclusion criteria included: (1) patients with acute or chronic infectious diseases; (2) patients with severe systemic diseases, such as cardiovascular, cerebrovascular, hepatic, renal, or hematologic disorders; (3) patients with malignant tumors or psychiatric disorders; (4) pregnant or lactating women; and (5) patients with joint infections. The healthy control group was labeled as S0, the active RA group as S1, and the stable RA group as S2. Four patient samples from each group were selected for sequencing for proteomics analysis, and the analysis workflow is shown in Figure 1. This study was approved by the Ethics Committee of the First People's Hospital of Nantong (approval no. 2024KT078). The research was conducted in accordance with the Good Clinical Practice (GCP) guidelines and the Declaration of Helsinki.

### 2.2 Serum Collection and Protein Extraction

Within 3 days of participant enrollment, 2.0–3.0 mL of venous blood was collected from each subject in the morning after overnight fasting. The samples were centrifuged at 3000 rpm for 10 min at 4°C, and the supernatant serum was transferred into Eppendorf tubes. The serum samples were stored at −80°C until analysis. Before the experiment, the samples were thawed on ice, then centrifuged at 12,000 × *g* for 10 min at 4°C to remove cellular debris. The supernatant was transferred to a new tube, and protein concentration was measured using a BCA Protein Assay Kit.

### 2.3 Trypsin Digestion

A total of 50 μL of the high‐speed centrifuged blood sample was added to pre‐washed magnetic nanomaterials (PTM‐00F13303, PTM‐biolab; silica‐coated Fe_3_O_4_ nanoparticles, 200 nm diameter) and incubated on a thermomixer at 1200 rpm, 37°C for 1 h. After incubation, the magnetic beads were washed three times with a wash buffer (50 mM ammonium bicarbonate, pH 8.0). Subsequently, 70 μL of digestion buffer (50 mM ammonium bicarbonate with 10% acetonitrile, pH 8.0) was added to the beads, mixed thoroughly, and heated at 95°C for 10 min. After cooling to room temperature, 20 ng/μL trypsin was added, and digestion was carried out overnight at 37°C. After digestion, dithiothreitol (DTT) was added to achieve a final concentration of 5 mM, and the samples were reduced to 56°C for 30 min. Subsequently, iodoacetamide (IAM) was added to a final concentration of 11 mM, and the samples were incubated at room temperature in the dark for 15 min. Salt removal was performed according to the C18 ZipTip protocol, and the samples were vacuum‐dried and prepared for liquid chromatography–tandem mass spectrometry (LC–MS/MS) analysis.

### 2.4 LC–MS/MS Analysis

Peptides were dissolved in mobile phase A and separated using an EASY‐nLC 1200 ultra‐high‐performance liquid chromatography (UHPLC) system. Mobile phase A consisted of water containing 0.1% formic acid and 2% acetonitrile, while mobile phase B contained 0.1% formic acid and 90% acetonitrile. The liquid chromatography gradient was as follows: 0–16 min, 6%–20% B; 16–24 min, 20%–32% B; 24–27 min, 32%–80% B; and 27–30 min, 80% B, with a flow rate of 500 nL/min. After separation by the UHPLC system, the peptides were ionized via the NSI ion source and analyzed using an Orbitrap Exploris 480 mass spectrometer. The ion source voltage was set to 2100 V, and both precursor ions and their fragments were detected and analyzed using the high‐resolution Orbitrap. The scan range for the MS1 spectrum was 350–1050 *m/z* with a resolution of 30,000. The MS2 scan range started at 200 *m/z*, with a resolution of 45,000. Data were collected using data‐independent acquisition (DIA), where multiple *m/z* windows were followed by peptide ions entering the HCD collision cell. Fragmentation energies of 25%, 30%, and 35% were applied for the MS2 analysis. To optimize mass spectrometer efficiency, automatic gain control (AGC) was set to 3E6, and the maximum injection time was set to Auto.

### 2.5 Database Query

#### 2.5.1 Spectral Library Construction

DDA data were processed using Spectronaut (v.18) software in conjunction with the Pulsar search engine. The tandem mass spectrometry sequence database used was Homo_sapiens_9606_SP_20231220. The Fasta file, containing 20,429 entries, was combined with a reverse decoy database. The maximum number of missed cleavages was set to 2. Carbamidomethylation of cysteine (Cys) was specified as a fixed modification, while protein N‐terminal acetylation and methionine (Met) oxidation were designated as variable modifications. The false discovery rate (FDR) for proteins, peptides, and PSMs was adjusted to < 1%. The constructed spectral library was imported into Spectronaut (v.18) software, where retention times were predicted using nonlinear calibration. DIA data were subsequently used for searches.

#### 2.5.2 Disease‐Related Database Query

Pyroptosis‐related target genes were retrieved from the GeneCards database (https://www.genecards.org/). The filtering criterion for database searches was a relevance score ≥ 1.

### 2.6 Gene Ontology (GO) Functional Annotation

GO functional annotation was performed using Thermo Proteome Discoverer 2.1 software for protein identification. Relative quantification was conducted using the peak areas of the *m/z* ions (S0114, S1115, and S2117), with *m/z* S0 as the control. The ratios S1:S0 and S2:S0 were calculated, and proteins with a fold change (FC) ≥ 1.5 (upregulated) or ≤ 0.7 (downregulated) were selected for further analysis. GO functional annotation was then applied to the identified serum proteins. *p* values were adjusted for multiple testing using the Benjamini–Hochberg FDR correction. Functional terms with an FDR < 0.05 were considered statistically significant.

### 2.7 Kyoto Encyclopedia of Genes and Genomes (KEGG) Pathway Annotation

Protein pathways were annotated using the KEGG pathway database. Protein identification was performed via BLAST (blastp, value ≤ 1e−4), and the annotation for each sequence was based on the top‐scoring alignment. Pathway enrichment significance was assessed using FDR‐adjusted *p* values (FDR < 0.05).

### 2.8 Clusters of Orthologous Groups/Eukaryotic Orthologous Groups (COG/KOG) Annotation

COG refers to proteins believed to have evolved from a common ancestor. Proteins within a COG are considered to be vertically inherited orthologs, typically maintaining the same function as the ancestral protein. COGs are classified into two categories: prokaryotic and eukaryotic. The prokaryotic database is commonly referred to as the COG database, while the eukaryotic database is known as the KOG database. Eggnog provides more comprehensive species classification and more homologous protein sequences than the NCBI COG database. It also includes phylogenetic tree construction and functional annotation for each homologous gene cluster.

### 2.9 Protein Functional Enrichment

Fisher's exact test was used to analyze the functional enrichment of DEPs, with the identified proteins serving as the background. Functional terms with an FC > 1.5 and a *p* value < 0.05 were considered statistically significant.

### 2.10 Clustering Based on Enrichment

Further hierarchical clustering was performed based on the functional classification of DEPs (e.g., GO, Domain, KEGG pathways, Reactome, and WikiPathways). First, all categories and their corresponding *p* values from the enrichment analysis were compiled. Categories with a *p* value < 0.05 in at least one cluster were selected for further analysis. The filtered *p* value matrix was then transformed using the *x* = −log_10_(*p* value) function. Subsequently, one‐way hierarchical clustering (Euclidean distance, average linkage) was applied to *p* values. Cluster members were visualized using heatmaps generated by the “Heatmap” function in the “Complex Heatmap” R package.

### 2.11 Protein–Protein Interaction (PPI) Network

DEPs were queried in the STRING database to identify PPIs. Only interactions between proteins in the search dataset were considered, excluding external candidates. STRING defines a “confidence score” to measure the reliability of interactions. Interactions with a confidence score > 0.7 (high confidence) were retained, as this threshold is commonly used to minimize false‐positive interactions while maintaining network connectivity. Sensitivity analyses using thresholds of 0.5 and 0.9 showed consistent identification of core hub proteins (including PCSK9, FN1, and APOE), supporting the robustness of the network. The interaction network was visualized using the “Vis Network” R package.

### 2.12 Enzyme‐Linked Immunosorbent Assay (ELISA)

The following human ELISA kits were purchased: PCSK9 (catalog no. EH0251‐HS), Caspase‐1 (catalog no. EH0595), GSDMD (catalog no. EH8956), NLRP3 (catalog no. EH4202‐HS), IL‐1β (catalog no. EH0185), IL‐1β (catalog no. EH0185), and IL‐18 (catalog no. EH0011). All kits were obtained from Fine Test. Experiments were conducted according to the manufacturer's instructions, and all assays were performed in triplicate to ensure reproducibility.

### 2.13 Statistical Analysis

Statistical analyses were performed using SPSS version 23.0. Graphical representations of the data were generated using GraphPad Prism 8.0. Each experiment was repeated at least three times to confirm the reproducibility of the results. Data are expressed as mean ± standard deviation (*X* ± *S*). For comparisons between the two groups, the Student's *t*‐test was used. A one‐way analysis of variance (ANOVA) was applied for comparisons among multiple groups. Correlation analysis was conducted using the appropriate method, such as Pearson or Spearman correlation. Pearson's correlation was used for data with a normal distribution, while Spearman's correlation was used for non‐normally distributed data. A *p* value of < 0.05 was considered statistically significant.

## 3 Results

To systematically identify pyroptosis‐related biomarkers in RA, we first performed high‐throughput LC–MS/MS proteomic profiling on serum samples from patients with RA and healthy controls. Differential expression analysis identified candidate proteins, which were subsequently analyzed via bioinformatics (GO, KEGG, and PPI) to prioritize key pathways and hub proteins. Based on this pipeline, PCSK9 emerged as a central node in the pyroptosis network and was selected for further validation using ELISA to confirm its association with RA pathogenesis.

### 3.1 Baseline Characteristics of Study Participants

The baseline demographic and clinical characteristics of the participants are summarized in Table 1. A total of 100 individuals were enrolled, including 50 healthy controls (S0), 35 patients with stable RA (S2), and 15 patients with active RA (S1). No significant differences were observed among the three groups in terms of age, gender distribution, or body mass index (BMI) (all *p* > 0.05), indicating that the groups were well‐matched for these potential confounding factors.

As per the predefined criteria (DAS28‐CRP > 3.2 for active RA), patients in the active RA group had significantly higher DAS28‐CRP scores than those in the stable RA group (3.97 ± 0.93 vs. 1.86 ± 0.81, *p* < 0.001). Both RA groups exhibited classic features of systemic inflammation, with significantly elevated levels of ESR and CRP, increased neutrophil percentage, decreased lymphocyte percentage, and lower hemoglobin levels compared to healthy controls (all *p* < 0.05). Notably, markers of humoral immune activation were also heightened in patients with RA, as evidenced by significantly increased levels of IgG, IgA, C3, and a higher proportion of CD19^+^ B cells (all *p* < 0.05), while the proportion of CD16^+^CD56^+^ NK cells was reduced.

Metabolic alterations were observed in patients with RA, including elevated fasting glucose, HbA1c, and total cholesterol levels compared to controls (all *p* < 0.05). Uric acid levels were significantly lower in the active RA group. No significant differences were found between the RA groups and controls regarding liver and kidney function tests, most lipid parameters, tumor markers, thyroid function, or the proportions of major T cell subsets (CD3^+^, CD4^+^, and CD8^+^). Medication profiles were similar between the stable and active RA groups.

### 3.2 Quality Control (QC) Analysis Confirms the Good Quality and Distinct Grouping of RA Sample Data

**FIGURE 2:**
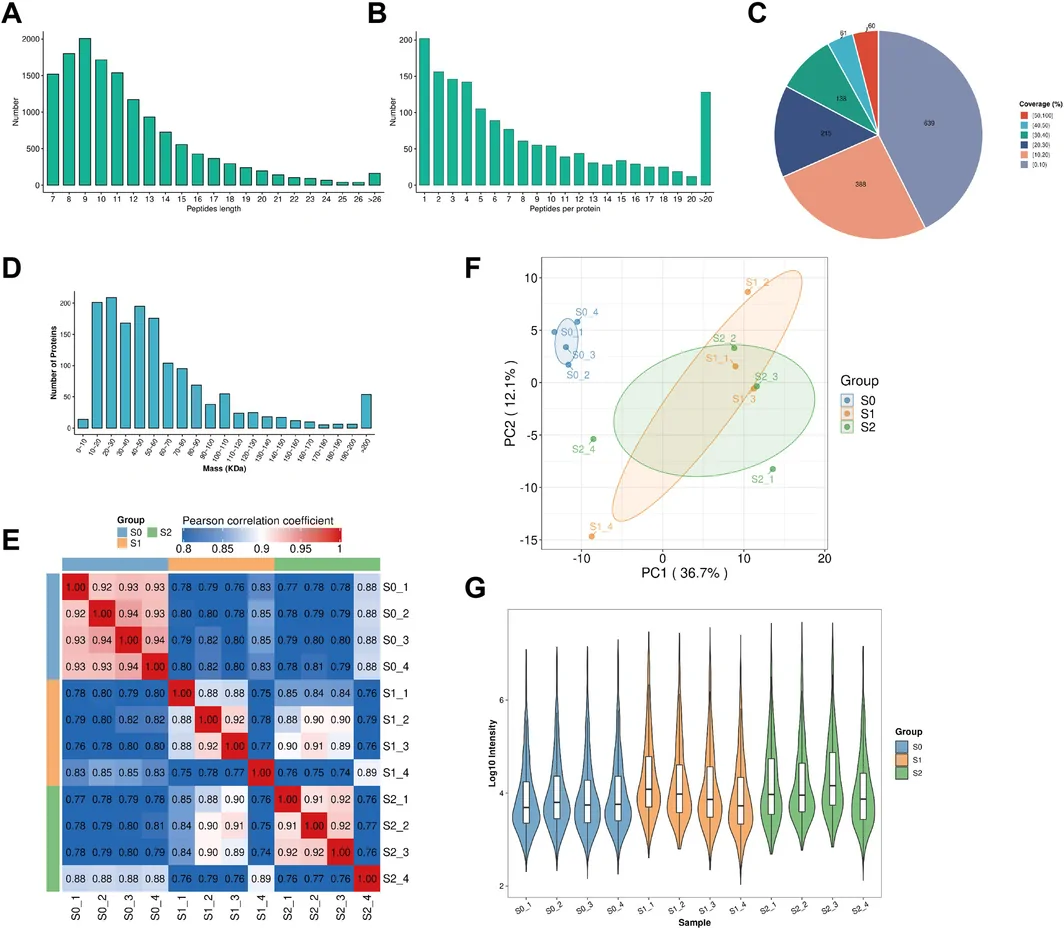
QC of proteomics data. *Note:* (A) Peptide length distribution across samples from different groups. (B) Peptide count distribution across samples from different groups. (C) Protein coverage distribution across samples from different groups, with protein coverage positively correlated with protein abundance in the samples. (D) Protein molecular weight distribution across samples from different groups. (E) Heatmap of pairwise PCC calculated from intensity values of all samples. Darker red indicates a higher correlation (closer to 1), signifying a more substantial similarity between the two samples. (F) PCA plot, where the *x*‐ and *y*‐axes represent the explained variance of PC1 and PC2, respectively. Higher values indicate greater explained variance. Clustering within groups reflects the reproducibility of samples, with replicates from the same group tending to cluster together. (G) Violin plot showing intensity values after Log_10_ transformation. The *x*‐axis represents the samples, while the *y*‐axis represents the transformed intensity values. Violin plot colors indicate different groups. The inner box plot represents the interquartile range (IQR), which contains the middle 50% of the data. The outer kernel density plot shows the probability distribution of the values. Larger kernel density areas correspond to higher probabilities of specific values, allowing a rough comparison of data distribution variability within and between groups.

To identify potential biomarkers for RA, we first conducted QC assessments of the acquired data to ensure the scientific validity and accuracy of the results. We evaluated several parameters, including peptide length distribution, peptide count distribution, protein coverage, and protein molecular weight distribution. The results showed that the peptides were predominantly 7–20 amino acids long, consistent with the typical patterns observed in enzymatic digestion and mass spectrometry fragmentation. Most proteins corresponded to more than two peptides, and most proteins had less than 30% coverage. The molecular weights of the identified proteins varied across different stages and were evenly distributed (Figure 2A–D). These findings suggest that the sequencing data are of high quality and suitable for subsequent analysis.

Next, we assessed the statistical consistency of technical replicates. The Pearson correlation coefficient (PCC) analysis indicated significant differences in correlation between the S0, S1, and S2 groups (Figure 2E). Principal component analysis (PCA) further revealed a clear distinction between normal and RA samples (Figure 2F), confirming that the samples from the different groups were well separated. Additionally, we examined the distribution and variability of protein intensity values between different samples. The results showed that the dispersion of data both within and between groups was relatively consistent (Figure 2G), further indicating the high quality of the samples.

### 3.3 Quantitative Screening Reveals Differential Protein Expression Profiles in Patients With RA

**FIGURE 3:**
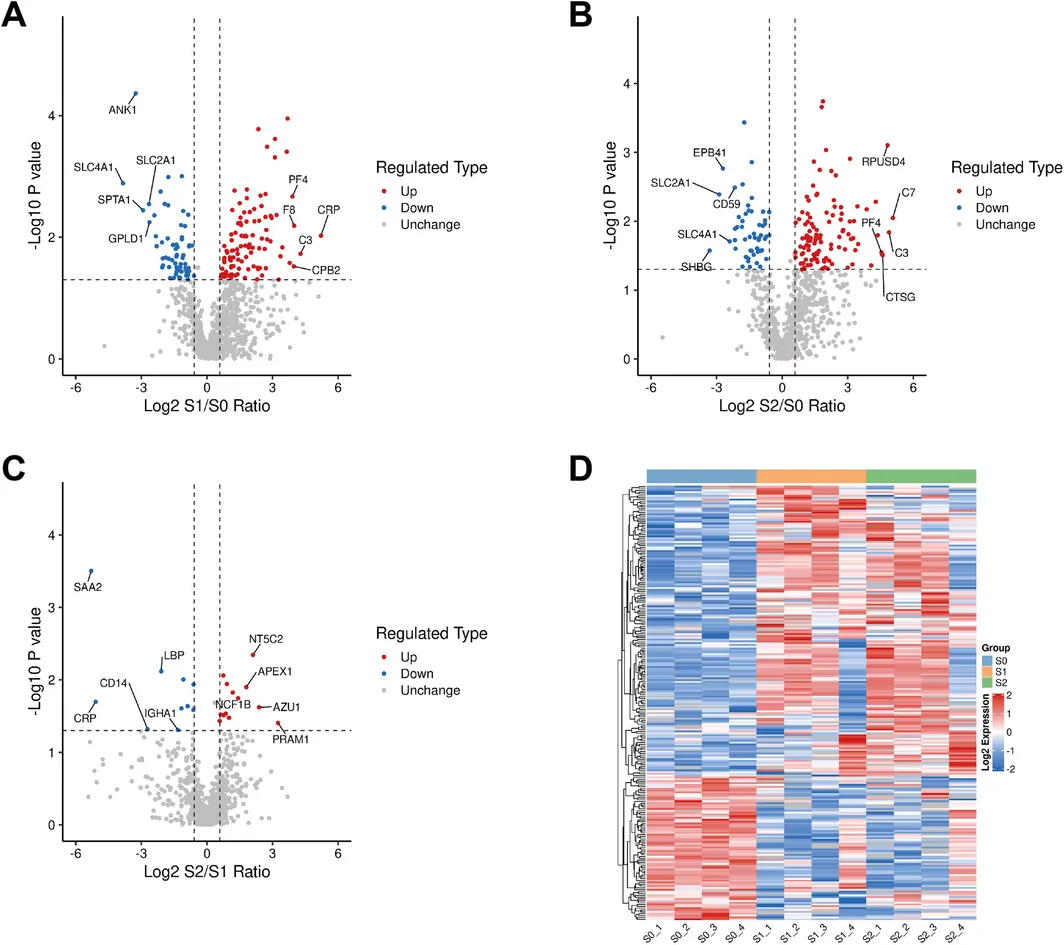
Differential protein quantification across groups. *Note:* (A–C) Volcano plots for S1/S0, S2/S0, and S2/S1 comparisons. The *x*‐axis represents the log_2_‐transformed ratio values, and the *y*‐axis represents the −log_10_‐transformed *t*‐test *p* values. Red points indicate significantly upregulated proteins, blue points indicate significantly downregulated proteins, and gray points indicate no significant differences. (D) Clustering analysis of the relative expression levels of differential proteins. Each row represents a different protein, and each column represents a sample. Red indicates high expression, blue indicates low expression, and gray indicates proteins that could not be quantified in the corresponding samples.

Subsequently, to identify DEPs, we conducted a quantitative differential analysis comparing the S0 group with the S1 and S2 groups and the S1 group with the S2 group. The FC was calculated as the ratio of the mean relative quantification values between the two groups. A *t*‐test was performed on the relative quantification values to assess the significance of the differences, with corresponding *p* values calculated for each comparison. The results showed that proteins with an FC greater than 1.5 were considered significantly upregulated, while those with an FC smaller than 1/1.5 were considered significantly downregulated. Specifically, 105 proteins were upregulated and 61 proteins downregulated in the S1/S0 comparison; 115 proteins were upregulated and 52 proteins downregulated in the S2/S0 comparison; and 13 proteins were upregulated and 11 proteins downregulated in the S2/S1 comparison (Figure 3). These findings indicate that patients with RA exhibit widespread dysregulation of protein expression compared to healthy individuals, whereas the pathological differences between the active and stable phases are relatively limited.

### 3.4 Proteomic Analysis Reveals Key Inflammatory and Immune‐Related Pathway Enrichment in Patients With RA

To uncover the key pathogenic mechanisms of RA, we performed proteomic analysis and conducted GO and KEGG pathway enrichment analyses on DEPs between patients with RA (S1 and S2 groups) and normal controls.

**FIGURE 4:**
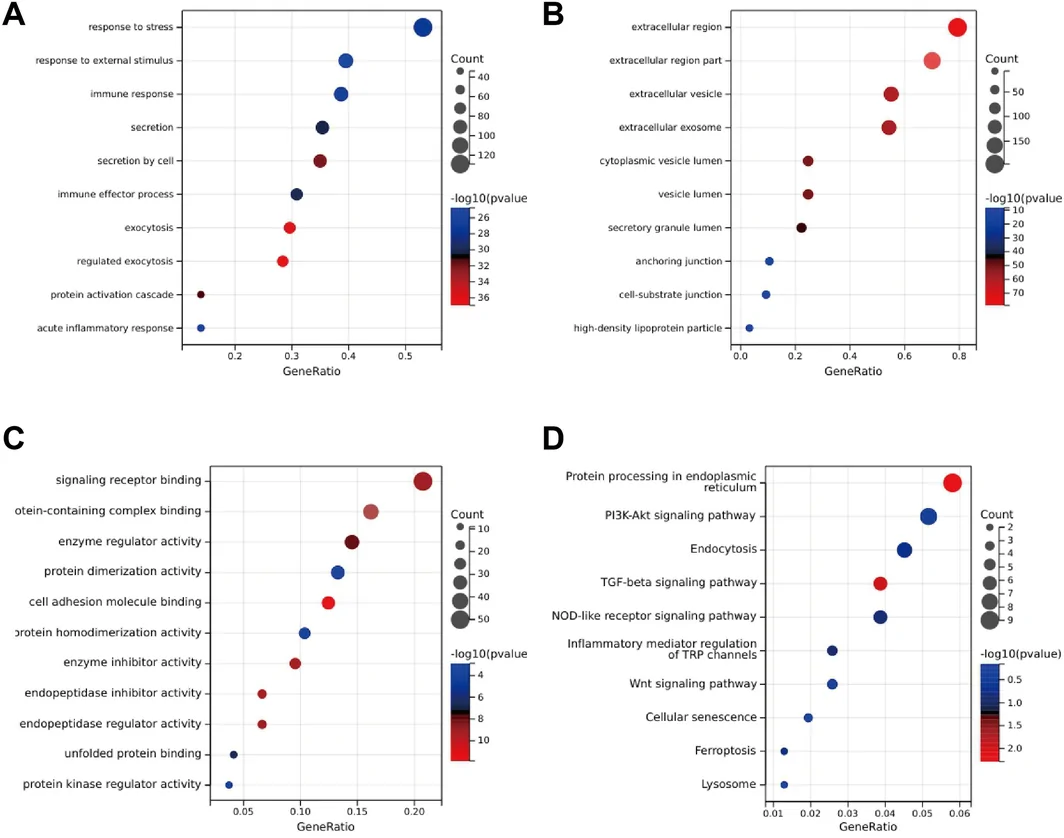
Enrichment analysis reveals key biological pathways in RA progression. *Note:* (A–C) GO enrichment results for differential proteins between S0 and the S1/S2 groups, including BP, CC, and MF. (D) KEGG enrichment analysis results for differential proteins between the S0 and S1/S2 groups.

GO enrichment analysis revealed that DEPs in patients with RA were predominantly enriched in key biological processes (BPs) related to immune response, inflammatory regulation, and secretion. In the BP category, these proteins were significantly enriched in immune‐ and inflammation‐associated pathways such as response to stress, response to external stimulus, immune response, immune effector process, and acute inflammatory response, as well as in processes related to protein secretion and signal transduction, including secretion, exocytosis, and protein activation cascade (Figure 4A). These results suggest that these proteins may contribute to RA pathogenesis by modulating immune responses and cytokine secretion. In the cellular component (CC) category, the proteins were highly enriched in extracellular structures such as the extracellular region, extracellular vesicle, extracellular exosome, cytoplasmic vesicle lumen, and secretory granule lumen (Figure 4B), indicating that vesicle‐mediated intercellular communication may play a central role in the RA‐associated immune network. Regarding the molecular function (MF) category, these proteins were significantly enriched in terms such as signaling receptor binding, protein‐containing complex binding, enzyme regulator activity, protein dimerization activity, and cell adhesion molecule binding (Figure 4C). These functions are involved in signal recognition, PPIs, and enzymatic regulation, suggesting that these proteins may influence RA pathophysiology by modulating immune signaling pathways and the formation of protein complexes.

KEGG pathway enrichment analysis revealed that the DEPs were significantly enriched in key pathways, including the NOD‐like receptor signaling pathway, NF‐kB signaling pathway, and cytokine‐cytokine receptor interaction (Figure 4D). Notably, both the NOD‐like receptor and NF‐kB signaling pathways play crucial roles in the immune‐inflammatory response in RA. The NOD‐like receptor signaling pathway is associated with the NLRP3 inflammasome, while activation of the NF‐kB pathway triggers the release of inflammatory cytokines, further promoting immune responses and joint damage [22]. The cytokine‐cytokine receptor interaction pathway is closely linked to immune system function and may enhance chronic inflammation in RA by regulating immune cell activity and recruitment. The TNF signaling pathway was also significantly enriched, suggesting that DEPs involved in TNF signaling may contribute to RA's immune mechanisms by promoting inflammation [23].

### 3.5 Identification of Key Regulatory Genes Associated With Pyroptosis in RA Pathogenesis

In the pathway enrichment analysis, we identified that inflammatory responses, particularly those related to the NLRP3 inflammasome, appear to be a critical component of RA pathogenesis. Pyroptosis, a programmed cell death triggered by inflammasome activation, is typically associated with intense inflammatory responses. Pyroptosis promotes the activation of inflammasomes (such as NLRP3) within cells, releasing pro‐inflammatory cytokines (e.g., IL‐1β and IL‐18), further exacerbating both local and systemic inflammation. Previous studies have reported that pyroptosis is a key mechanism driving persistent inflammatory responses in immune cells and joint damage [24]. Thus, the interaction between inflammation and pyroptosis may represent one of the core pathways in RA pathogenesis.

**FIGURE 5:**
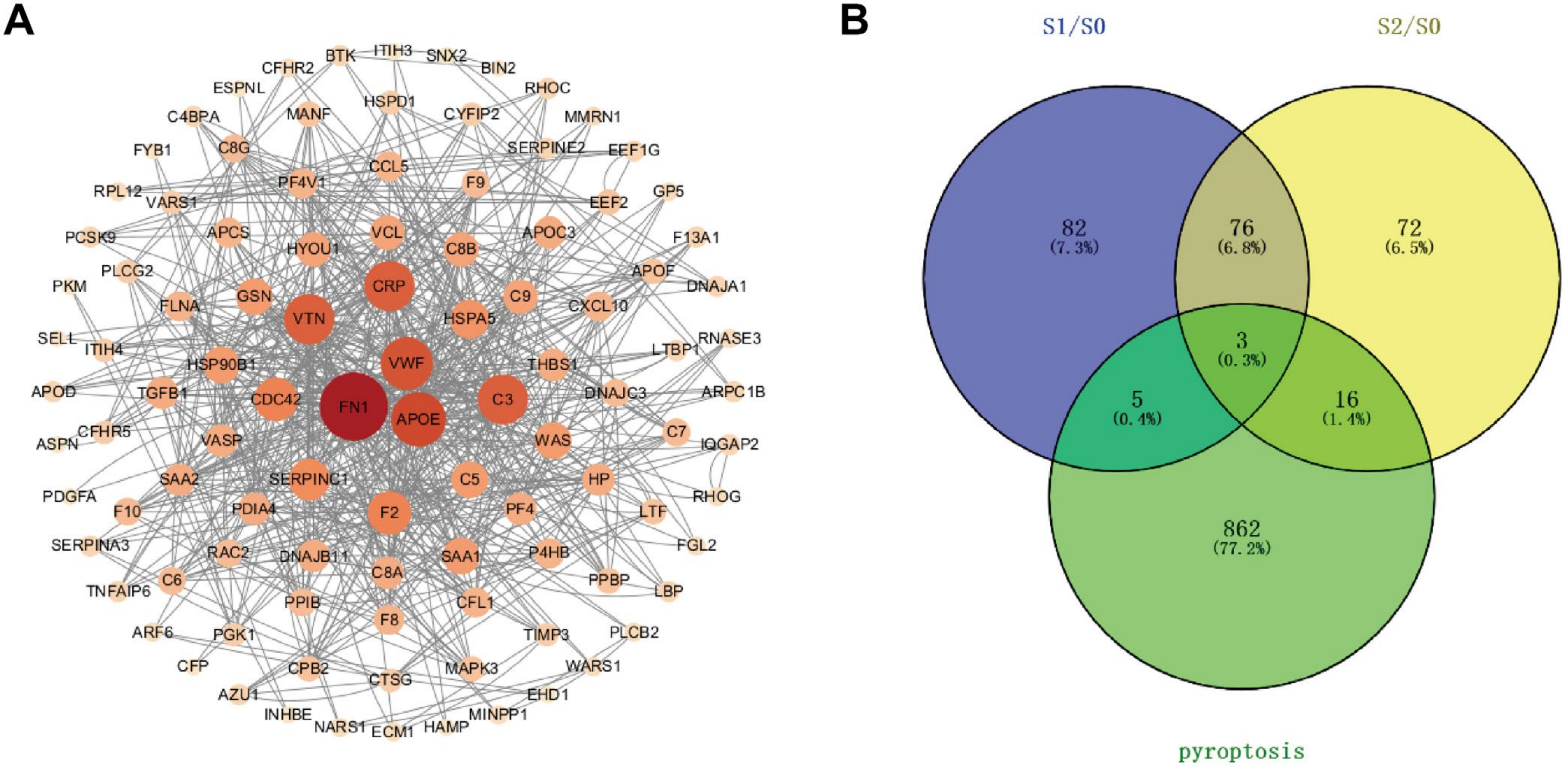
Key biomarker identification related to pyroptosis in RA progression. *Note:* (A) PPI network for differentially upregulated proteins in the S1 and S2 groups compared to S0. Circle size represents the degree of centrality, and lines indicate gene interactions. (B) Intersection of upregulated genes and pyroptosis‐related genes.

We used the STRING database to construct a PPI network comparing the S1 and S2 groups with the S0 groups to identify further biomarkers associated with pyroptosis in RA. The analysis revealed that genes such as FN1, APOE, and VMF occupy central positions in the network (Figure 5A). These proteins are closely linked to both inflammatory and immune responses. Additionally, a search of the GeneCards database for pyroptosis‐related genes yielded three core genes—SERPINC1, PCSK9, and H4C1—by intersecting the upregulated genes from the S1/S0 and S2/S0 comparisons (Figure 5B). These results suggest that SERPINC1, PCSK9, and H4C1 may be biomarkers of pyroptosis‐related pathways in RA progression.

### 3.6 ELISA Validation Reveals That PCSK9 May Promote RA Development via the Pyroptosis Pathway

Studies have shown that PCSK9 may promote the activation of the NLRP3 inflammasome, thereby inducing the activation of Caspase‐1. Activated Caspase‐1 cleaves gasdermin D (GSDMD), leading to the formation of membrane pores that trigger pyroptosis and promote the release of pro‐inflammatory cytokines such as IL‐1β and IL‐18, which in turn amplify local inflammatory responses (PMID: 38886277). This pathway plays a particularly important role in RA, suggesting that PCSK9 may exacerbate disease progression by enhancing immune cell activation and inflammation.

**FIGURE 6:**
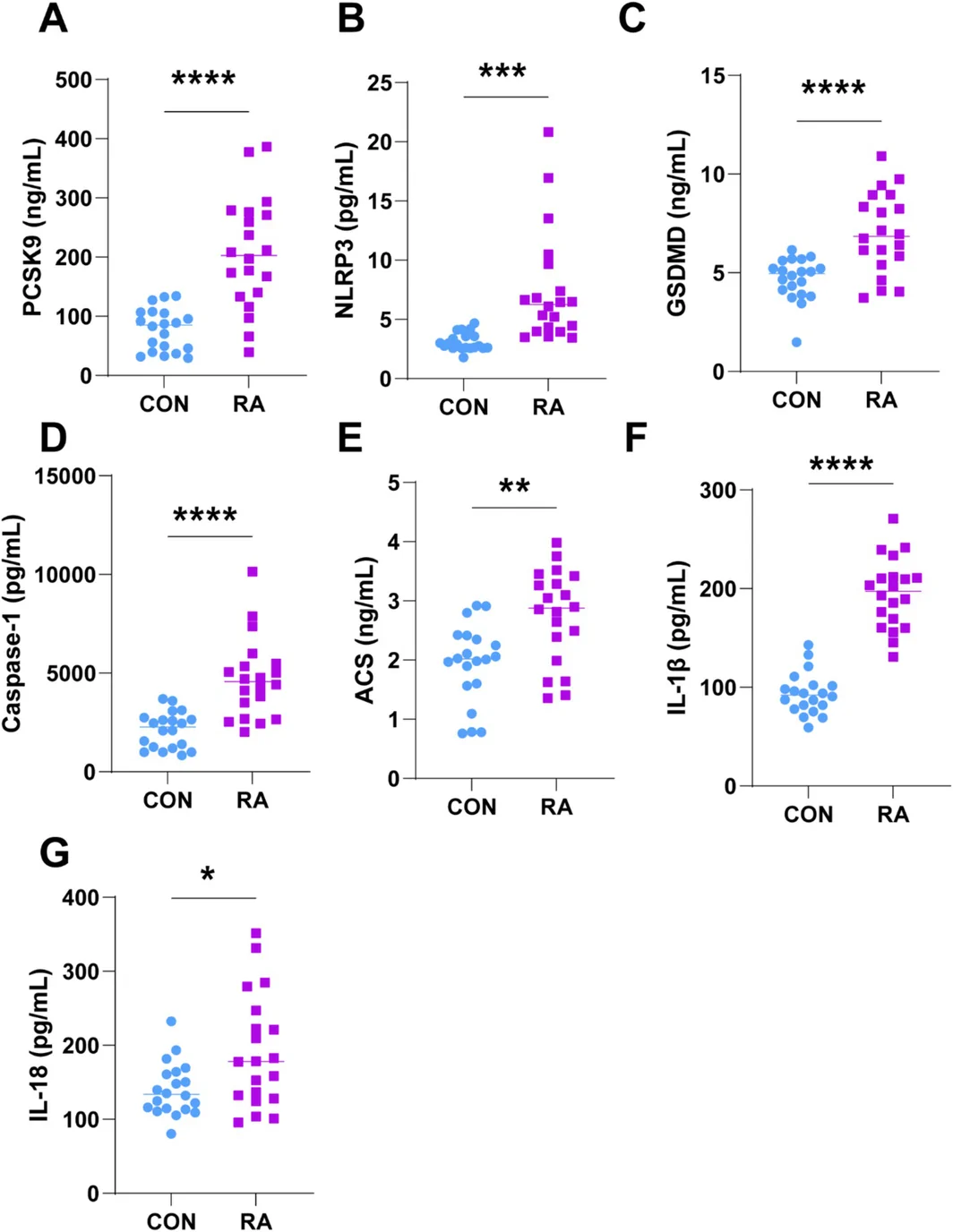
Analysis of PCSK9 and related protein expression levels in the serum of patients with rheumatoid arthritis (RA). *Note:* (A) ELISA detection of PCSK9 levels in the serum of patients with RA and healthy controls; (B–D) ELISA detection of NLRP3, GSDMD, and Caspase‐1 levels in the serum of patients with RA and healthy controls; (E–G) ELISA detection of AGS, IL‐1β, and IL‐18 levels in the serum of patients with RA and healthy controls. *p < 0.05, **p < 0.01, ***p < 0.001, and ****p < 0.0001.

To investigate the role of PCSK9 in RA pathogenesis, we performed ELISA assays to quantify the expression of PCSK9 and inflammasome‐related factors in the serum of patients with RA and healthy individuals. The results demonstrated that serum levels of PCSK9 and related inflammatory markers were significantly elevated in patients with RA compared to healthy controls. Specifically, PCSK9 levels were markedly increased in patients with RA (*p* < 0.0001). Key components of the NLRP3 inflammasome pathway, including NLRP3 (*p* = 0.0002) and Caspase‐1 (*p* < 0.0001), were significantly upregulated, as was the pyroptosis execution protein GSDMD (*p* < 0.0001). Expression of the pyroptosis‐associated molecule AGS was also significantly higher in the RA group (*p* = 0.0012). Notably, levels of the pro‐inflammatory cytokines IL‐1β (*p* < 0.0001) and IL‐18 (*p* = 0.0102) were significantly increased (Figure 6).

**FIGURE 7:**
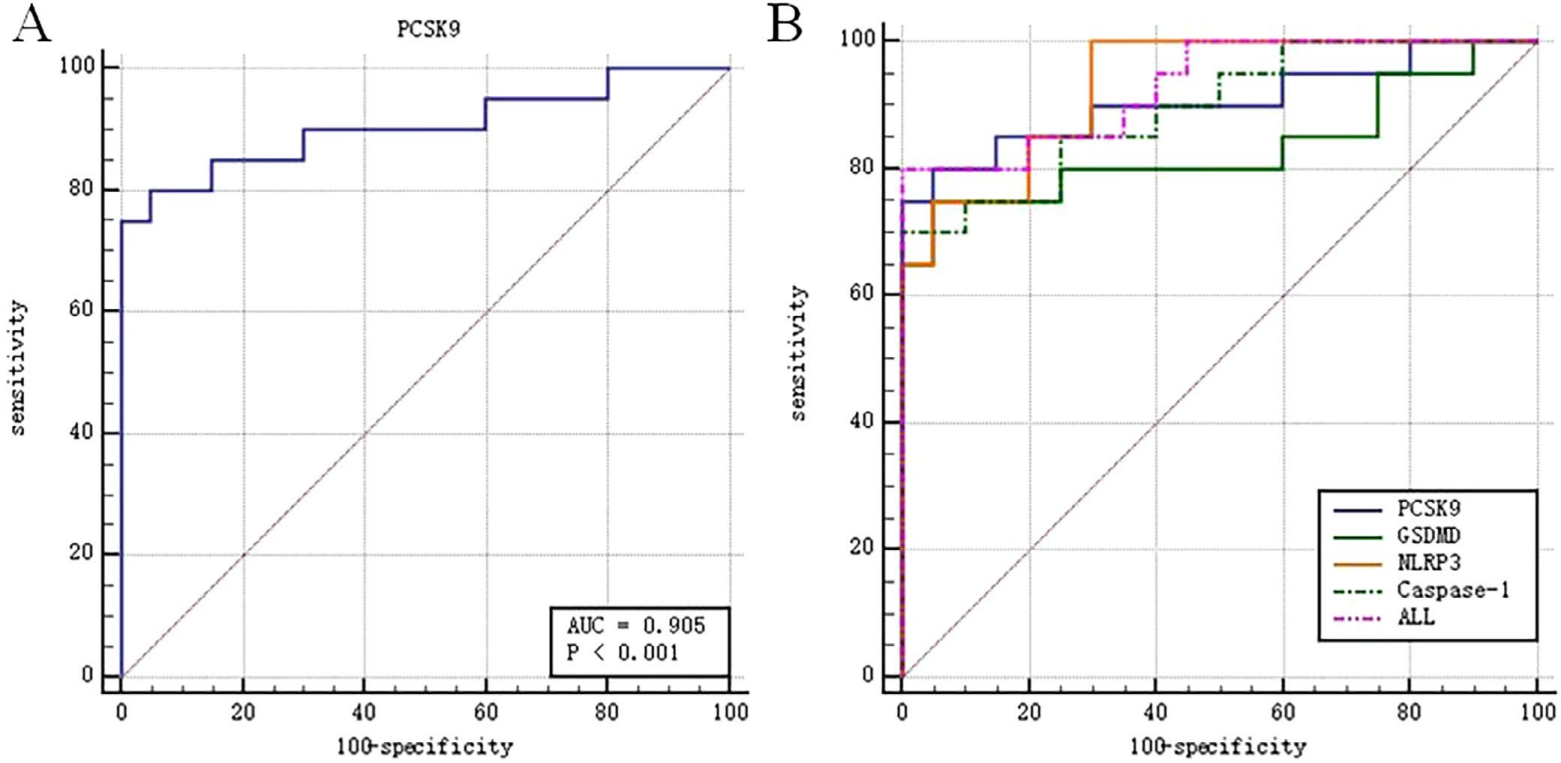
Diagnostic performance of serum biomarkers for rheumatoid arthritis (RA) detection. *Note:* (A) Receiver operating characteristic (ROC) curve showing the diagnostic performance of PCSK9 alone in discriminating patients with RA from healthy controls. The area under the curve (AUC) is 0.905 (95% CI: 0.770–0.975, *p* < 0.001), with a sensitivity of 80.00% and specificity of 95.00% at the optimal cutoff. (B) ROC curve analysis of the combined pyroptosis‐related biomarker panel for RA diagnosis. ROC curve demonstrating the diagnostic performance of a panel comprising PCSK9, NLRP3, Caspase‐1, and GSDMD. The combined biomarkers achieved an AUC of 0.930 (95% CI: 0.803–0.986, *p* < 0.001) with 100.00% specificity and 80.00% sensitivity.

The diagnostic potential of serum PCSK9 was evaluated using ROC curve analysis (Figure 7A). PCSK9 alone demonstrated excellent diagnostic accuracy for RA, with an AUC of 0.905 (95% CI: 0.770–0.975), sensitivity of 80.00%, and specificity of 95.00% at the cutoff > 132.50.

We further explored whether combining PCSK9 with other pyroptosis‐related markers could improve diagnostic performance. A panel comprising PCSK9, NLRP3, Caspase‐1, and GSDMD achieved the highest AUC of 0.930 (95% CI: 0.803–0.986) with 100% specificity, although the improvement over PCSK9 alone was marginal (AUC increase: 0.025) (Figure 7B).

Collectively, these findings suggest that PCSK9 promotes the release of IL‐1β and IL‐18 by activating the NLRP3/Caspase‐1/GSDMD‐mediated pyroptotic pathway, thereby contributing to the inflammatory cascade in RA.

## 4 Discussion

RA is a typical chronic autoimmune disease characterized by inflammation, pain, swelling, and functional impairment of the joints. As the disease progresses, it may lead to irreversible joint damage. Although the precise pathogenesis of RA remains unclear, studies have indicated that excessive immune activation, abnormal cytokine release, and programmed cell death play critical roles in the onset and progression of the disease [25]. In recent years, pyroptosis, a form of programmed cell death, has garnered increasing attention. Pyroptosis is closely associated with various immune‐mediated inflammatory diseases and may play a pivotal role in the immune activation and joint damage observed in RA [26]. This study utilized proteomic analysis to investigate the potential role of pyroptosis‐related biomarkers in the pathogenesis of RA. Specifically, it highlights that PCSK9 may contribute to RA by activating the NLRP3 inflammasome, thereby mediating pyroptosis and influencing the key mechanisms underlying RA. These findings provide new therapeutic and preventive targets for RA.

This study employed LC–MS/MS to conduct an in‐depth analysis of serum samples from patients with RA, identifying several DEPs associated with pyroptosis, immune response, and inflammation pathways. Notably, in patients with active RA, the expression of pyroptosis‐related proteins (such as NLRP3, Caspase‐1, GSDMD, and IL‐18) was significantly elevated, suggesting that pyroptosis may play a crucial role in the immune response of RA. GO functional annotation and KEGG pathway analysis revealed that these DEPs were significantly enriched in pathways related to pyroptosis, immune response, and cytokine signaling. In particular, changes in several key proteins during the activation of the NLRP3 inflammasome and subsequent signaling events indicate that pyroptosis may be a key mechanism in the immune response of RA. Further PPI network analysis identified novel molecular biomarkers associated with RA pathogenesis, including SERPINC1, PCSK9, and H4C1. These proteins may regulate immune responses and mediate inflammatory responses through interactions with immune cells, ultimately promoting pyroptosis and exacerbating the clinical manifestations of RA.

Pyroptosis is a form of programmed cell death mediated by the NLRP3 inflammasome. In this process, activation of the NLRP3 inflammasome triggers the cleavage of Caspase‐1, which promotes the maturation and release of pro‐inflammatory cytokines, such as IL‐1β and IL‐18. These cytokines play a central role in immune responses by enhancing local inflammation, significantly driving the onset and progression of RA [27]. Our study reveals a marked increase in the expression of proteins such as NLRP3, Caspase‐1, and GSDMD in the serum of patients with RA, further confirming the critical role of pyroptosis in the immune pathology of RA. Notably, excessive activation of the NLRP3 inflammasome drives a strong inflammatory cascade through the release of IL‐1β and IL‐18, leading to the excessive recruitment and activation of immune cells. This sustained immune activation not only exacerbates clinical symptoms of RA, including joint swelling, pain, and morning stiffness, but also promotes persistent inflammation in the synovium, cartilage destruction, and bone erosion [28].

In recent years, PCSK9, a protein that regulates cholesterol metabolism, has garnered increasing attention for its potential role in regulating pyroptosis and its known functions. Pyroptosis is a form of programmed cell death that depends on activating the NLRP3 inflammasome, typically accompanied by Caspase‐1 activation and GSDMD‐mediated pore formation in the cell membrane. PCSK9 regulates this process through multiple mechanisms. First, it induces the expression of the NLRP3 inflammasome and its associated components (e.g., ASC and Caspase‐1) by activating the upstream NF‐κB signaling pathway, thereby providing the molecular basis for inflammasome assembly and activation [29, 30, 31]. Second, PCSK9 may modulate mitochondrial function, producing excessive reactive oxygen species (ROS), a key inducer of NLRP3 inflammasome activation. This oxidative stress not only promotes NLRP3 activation but also further enhances the efficiency of pyroptosis [32, 33, 34]. Notably, our ROC analysis revealed that PCSK9 alone exhibited strong diagnostic value (AUC = 0.905), supporting its potential as a standalone serum biomarker for RA detection. While the combination of four pyroptosis‐related markers (PCSK9, NLRP3, Caspase‐1, and GSDMD) achieved slightly higher accuracy (AUC = 0.930) and perfect specificity (100%), the modest improvement suggests that PCSK9 measurement alone may be sufficient for initial screening in clinical settings, balancing diagnostic performance with cost‐effectiveness and simplicity.

Furthermore, PCSK9 may regulate the balance of cell membrane lipid metabolism, altering the biochemical properties of the membrane and thereby enhancing GSDMD‐mediated pore formation. This efficiently releases intracellular substances such as IL‐1β and IL‐18 [35]. The release of these pro‐inflammatory cytokines plays a crucial role in amplifying pyroptosis. Through a paracrine mechanism, IL‐1β and IL‐18, key pro‐inflammatory cytokines, can activate neighboring immune cells, including macrophages and neutrophils. This further amplifies the local inflammatory response. The inflammatory‐pyroptosis feedback loop is a critical mechanism underlying the persistent inflammation and tissue destruction in synovial tissue in inflammatory diseases like RA [36, 37]. Notably, PCSK9's role in immune cells is particularly prominent. Its abnormal expression may exacerbate excessive immune cell activation and the overproduction of pro‐inflammatory cytokines, thereby enhancing NLRP3 inflammasome activation and Caspase‐1 activation. This contributes to a vicious cycle of inflammation and pyroptosis [38].

In addition, PCSK9 may indirectly influence the pyroptosis process by regulating cholesterol metabolism. The distribution and content of cholesterol in the cell membrane significantly impact the formation and stability of membrane pores. PCSK9, by degrading the low‐density lipoprotein receptor (LDLR), affects the transport and accumulation of cholesterol in the membrane. This may enhance the stability of GSDMD‐mediated membrane pore formation, thereby exacerbating the release of cellular contents. More importantly, disruptions in cholesterol metabolism could trigger metabolic inflammation, providing metabolic support for activating the inflammasome and amplifying pyroptosis [39]. In summary, PCSK9 may enhance the activation of the NLRP3 inflammasome through signaling pathways related to lipid metabolism in the cell membrane, thereby promoting pyroptosis. Pyroptosis releases large amounts of pro‐inflammatory cytokines, such as IL‐1β and IL‐18, which can further activate immune cells, creating a vicious cycle that exacerbates the immune‐inflammatory response in RA [40]. In addition to PCSK9, our PPI network identified other highly connected proteins, including fibronectin 1 (FN1) and apolipoprotein E (APOE). FN1 is a key extracellular matrix glycoprotein involved in cell adhesion, migration, and inflammation, and its overexpression has been linked to synovial fibrosis and joint destruction in RA. APOE, known for its role in lipid metabolism, also exhibits immunomodulatory functions and may influence inflammatory responses in autoimmune diseases. Although not the focus of this study, the strong connectivity of FN1 and APOE within the pyroptosis‐related network suggests potential crosstalk with the PCSK9‐NLRP3 axis, warranting further investigation into their synergistic roles in RA pathogenesis.

In this study, the three groups showed no significant differences in baseline characteristics such as age, gender, and body mass index (*p* > 0.05), indicating good comparability. Patients with RA exhibited typical systemic inflammatory features (e.g., elevated ESR and CRP, *p* < 0.05) and metabolic disturbances (e.g., increased blood glucose and lipids, *p* < 0.05), which hold clear pathophysiological relevance. However, most conventional inflammatory markers showed no significant differences between active and stable RA (*p* > 0.05), likely due to similar background antirheumatic drug regimens in both groups. This suggests the limited sensitivity of these indicators in distinguishing disease activity and underscores the necessity of exploring novel molecular biomarkers such as PCSK9.

Although this study suggests a potential link between PCSK9, pyroptosis, inflammation, and the pathogenesis of RA, several limitations remain. First, this study focused on serum samples due to their clinical accessibility, minimal invasiveness, and relevance for systemic biomarker discovery in RA. Serum reflects the systemic inflammatory and immune status, which is crucial for understanding RA as a systemic autoimmune disease. While synovial fluid or tissue may provide more direct insight into local joint pathology, serum biomarkers are advantageous for routine clinical screening and monitoring. Future studies should integrate synovial fluid proteomics to validate the local expression and role of PCSK9 and other pyroptosis‐related proteins within the joint microenvironment. Second, while this study identified several DEPs associated with RA through proteomics, functional validation of these proteins remains limited. To further confirm the role of PCSK9 in RA, subsequent studies should incorporate cell‐based experiments (e.g., PCSK9 overexpression/knockdown in synovial fibroblasts or macrophages) and animal models (e.g., collagen‐induced arthritis treated with PCSK9 inhibitors) to assess the specific mechanisms by which PCSK9 regulates the NLRP3 inflammasome and pyroptosis and its contribution to RA progression. Third, this study focused on protein expression changes in patients with RA without systematically analyzing the clinical characteristics or immune cell subsets. Future research should combine clinical data to investigate the relationship between PCSK9, pyroptosis biomarkers, and RA clinical manifestations. Fourth, the single‐center design and hospital‐based recruitment may introduce selection bias and limit the generalizability of our findings. Additionally, although we have provided medication profiles (Table 1), the use of DMARDs and biologics may confound serum protein levels; future multi‐center studies with larger, treatment‐naïve or medication‐stratified cohorts are needed to validate our results. Finally, the therapeutic potential of PCSK9 was only suggested through proteomic analysis in this study; further clinical intervention trials are necessary to validate the efficacy and safety of PCSK9 inhibitors in RA treatment. Therefore, future studies should explore the feasibility of targeting PCSK9 in RA therapy and assess its effectiveness and safety in a broader clinical context.

## 5 Conclusion

**FIGURE 8:**
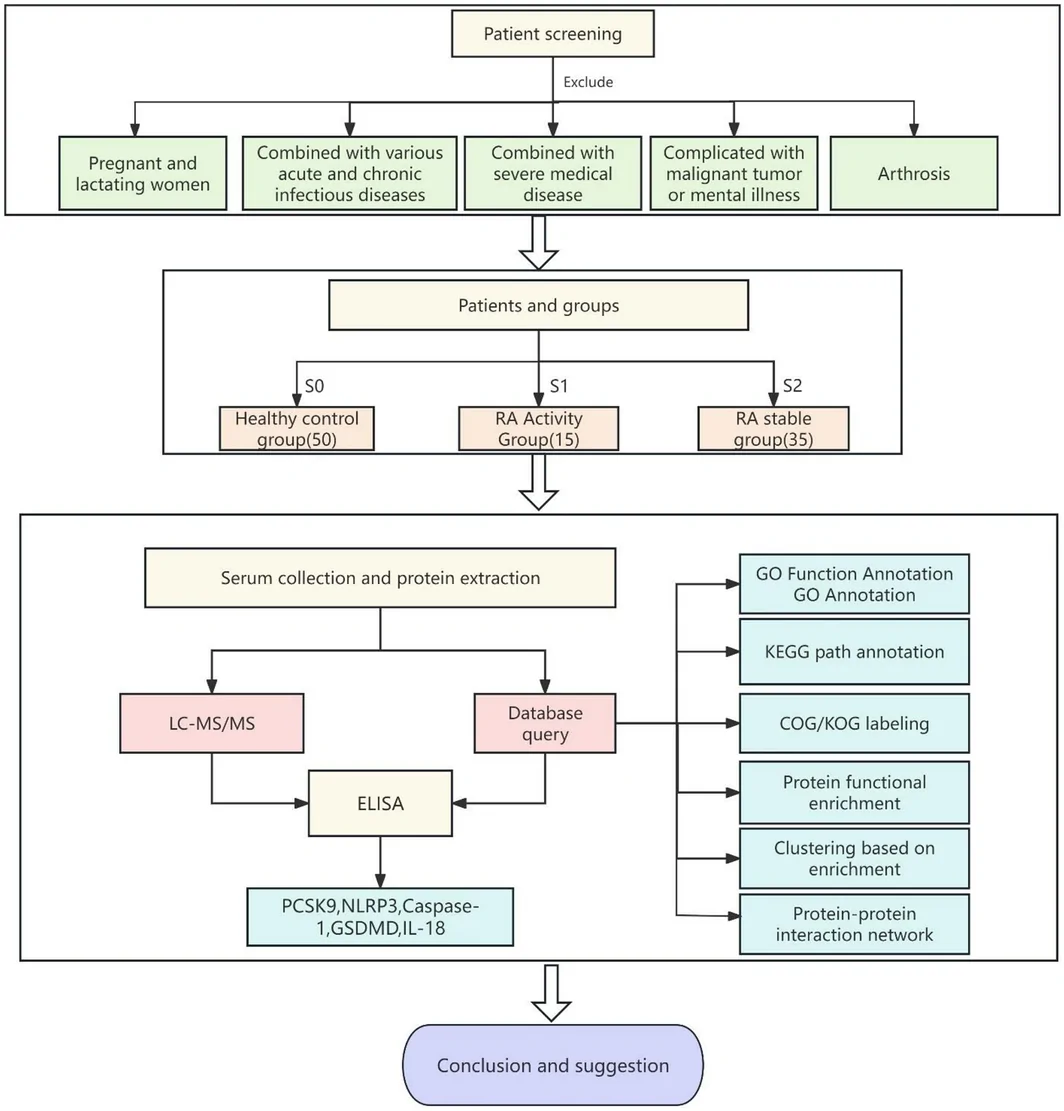
Research workflow diagram.

This study reveals the potential role of pyroptosis‐related biomarkers in the pathogenesis of RA through proteomic analysis, focusing on the key regulatory role of PCSK9 in RA immune responses and pyroptosis. DEPs identified using high‐throughput LC–MS/MS indicate that the activation of the NLRP3 inflammasome and its associated signaling pathways plays a crucial role in the immune response of RA. Moreover, the expression of pyroptosis‐related proteins, such as NLRP3, Caspase‐1, GSDMD, and IL‐18, is significantly elevated in the serum samples of patients with RA, suggesting that pyroptosis contributes to both the immune response and joint damage in RA. Notably, PCSK9, as a potential biomarker, may enhance the activation of the NLRP3 inflammasome by modulating immune cell function, thereby exacerbating the inflammatory response and pyroptosis process in RA, which further drives the pathological progression of the disease (see flowchart in Figure 8). This study provides new molecular targets for the early diagnosis and targeted therapy of RA and offers theoretical support for the potential application of PCSK9 in immune regulation and RA treatment.

## Funding

The study was supported by the Medical Innovation Application of Suzhou Science and Technology Bureau (SKY2023161), the Medical Research Project of Jiangsu Provincial Health and Wellness Commission (Z2022067), and Suzhou Industrial Park Oriental Huaxia Cardiovascular Health Institute–Natural Lipid‐Regulating Drug Evidence‐Based Research Fund Program (2023‐CCA‐NLD‐638).

## Ethics Statement

This study has been approved by the Ethics Committee of the First People's Hospital of Nantong (No. 2024KT078).

## Conflicts of Interest

The authors declare no conflicts of interest.

## Data Availability

The data that support the findings of this study are available from the corresponding author upon reasonable request.
